# Spatial distribution characteristics and pollution evaluation of soil heavy metals in Wulongdong National Forest Park

**DOI:** 10.1038/s41598-024-58259-5

**Published:** 2024-04-17

**Authors:** Xiaolong Chen, Hongfeng Zhang, Cora Un In Wong

**Affiliations:** 1 Faculty of Humanities and Social Sciences, Macao Polytechnic University, Macao, China; 2 Department of Management, Henan Institute of Technology, Xinxiang, Henan China

**Keywords:** Forest soil, Heavy metal pollution, Spatial distribution, Pollution assessment, Wulongdong National Forest Park, Ecology, Environmental sciences

## Abstract

To scrutinize the spatial distribution attributes of soil heavy metal content and discern its pollution status within the expanse of Wulongdong National Forest Park, a meticulous investigation is imperative. Three altitude gradients of 900, 1000, and 1069 m were selected on the shady and sunny slopes of Wulongdong National Forest Park, and a total of 300 soil sample points were collected. Soil samples were collected in layers, and the contents of seven soil heavy metal elements, Cr, Cd, Hg, Ni, Se, As, and Pb, were measured. With regard to the national soil element background values, the single factor index method, Nemerow index method, and pollution load index were employed to undertake a thorough assessment of soil heavy metal pollution. (1) The contents of heavy metal elements Cr, Se, As, and Pb in the 0–20 cm soil layer of Wulongdong National Forest Park are lower than the national soil element background value and the Henan soil element background value; the Cd and Hg contents exceed the national soil element background value. The value and Henan soil element background value are 2.2 times and 2.92 times the national soil element background value, and 2.75 times and 9.5 times the Henan soil element background value respectively; Ni content is lower than the Henan soil element background value, but higher than the national soil element background value. The background value is 1.03 times its content. The coefficients of variation of the contents of seven heavy metal elements are all greater than 50%, among which Hg shows extreme variation, and the remaining six are highly variable. (2) In the same soil layer, the Cr and As contents are lower on sunny slopes than on shady slopes, and the contents of Pb, Ni, and Hg are generally higher on sunny slopes than on shady slopes. On sunny slopes, the contents of As, Cd, and Hg decrease with increasing altitude, and the Se content increases with increasing altitude; while on shady slopes, the contents of Cr, Se, and As decrease with increasing altitude, and Pb and Hg content increase with the increase of altitude; the content of heavy metal element As increases with the deepening of the soil layer on shady slopes, and the Hg content decreases with the deepening of the soil layer on sunny slopes. The contents of other heavy metal elements have no obvious regularity among different slope directions, altitudes and soil layers. (3) The single factor index evaluation results show that in the 0 ~ 20c soil layer and on the sunny slope, Hg is heavily polluted, Cd is moderately polluted, Ni is lightly polluted, and Cr, Se, As, and Pb are all non-polluted; On the shady slope, Cd and Hg are moderately polluted, and the other five heavy metal elements are in a non-polluting state. (4) The Nemerow index method evaluation results show that in the 0 ~ 20 cm soil layer, the soil on sunny slopes is significantly more polluted by heavy metals than on shady slopes, and the main pollutants are Ni, Cd and Hg. (5) In the 0 ~ 20 cm soil layer of Wulongdong National Forest Park, the three heavy metal elements Ni, Cd and Hg have reached pollution levels, of which Ni is slightly polluted, Cd and Hg are moderately or above polluted; the sunny slope soil is slightly polluted. Heavy metal pollution, no heavy metal pollution on shady slopes.

## Introduction

A considerable deluge of heavy metal contaminants infiltrates the soil through industrial byproducts, commonly denominated as the "three wastes". This instigates a conspicuously heightened concentration of heavy metals within the soil matrix^1^. This phenomenon poses a grave peril to the pristine quality of the urban ecological milieu. The arboreal domain within the urban ecosystem, commonly acknowledged as the urban forest, stands as the solitary subsystem that actualizes the inherent negative feedback mechanism of "absorbing pollution and generating renewal". Functioning as the paramount nexus and intermediary region for heavy metal pollutants within urban soil^2^, it manifests a salient defensive efficacy against the deleterious encroachment of heavy metal pollution. Urban forests are an important ecological barrier to protecting the urban environment^3^. Affected by urban microclimate, human activities, environmental pollution, and other factors, soil degradation is serious, which restricts the improvement of urban ecological service functions^4–6^. The forest is the largest ecosystem on land, and it has various ecological functions such as water conservation, soil and water conservation, air purification, pollution prevention, climate regulation, windbreaks, and sand fixation^7^. The soil stands as an integral facet within the arboreal ecosystem, embodying the substantive foundation that sustains vegetative life. The quality of the soil intricately shapes the ecological milieu of the forest, playing a momentous role in the overarching dynamics of its environmental equilibrium^8^. Concurrently, soil heavy metals exhibit the attributes of formidable degradation resistance, elevated toxicity, facile enrichment, and robust environmental tenacity. The rapid pace of urbanization, driven by human endeavors, has led to the infiltration of heavy metal pollutants into forest soil through numerous channels. These pathways encompass atmospheric deposition, discharge of industrial waste, application of agricultural chemical fertilizers, and pollution stemming from vehicular traffic. Collectively, these processes wield a profound impact, markedly disturbing the ecological equilibrium of the forest environment^9,10^.

As an intrinsic component and environmental influencer within the arboreal ecosystem, soil coordinates the distribution of water both internally and externally, acting as a discerning sieve. Additionally, it furnishes the requisite environmental milieu for the growth, maturation, and reproductive processes of the forest^11^. With the rapid development of China in the past few decades, activities such as smelting, mining, waste treatment, pesticide and fertilizer application, and vehicle exhaust emissions inevitably input heavy metals into urban and agricultural soils in various ways^12,13^. Metropolitan green spaces harbor the potential to mitigate both urban and regional environmental pollution, elevating the overall quality of the living milieu. China places significant emphasis on the prominence of urban green spaces and the discerning management of soil heavy metal pollution^14^.

The similarities and differences in soil heavy metal content between different altitudes and slope aspects may be controlled by multiple factors such as climate, topography, soil erosion^15–17^. This variance, in its essence, exerts a profound influence on the dispersion of contaminants within the soil. By delving deeply into these distinctions, we may glean a more precise comprehension of the functioning of these elemental components within the soil milieu. Differences in soil heavy metal content at different altitudes and slopes may be a reflection of differences in material circulation and migration in ecosystems^18^. On shady and sunny slopes, the diversity of vegetation types and coverage will also exert a unique influence on the adsorption and enrichment of heavy metals in the soil.

Therefore, in view of the different soil pollution problems caused by different altitudes and shady and sunny sides of forest green spaces, this study set up 300 sampling points at different altitude gradients on the shady and sunny slopes of Wulongdong National Forest Park to measure chromium (Cr), The contents of seven heavy metals: cadmium (Cd), mercury (Hg), nickel (Ni), selenium (Se), arsenic (As), and lead (Pb), to reveal the presence of heavy metals in the forest soil of Wulongdong National Forest Park under current environmental conditions. The singular factor index method, Nemerow index, and pollution load index methodologies were employed to comprehensively assess the pollution levels of soil heavy metals, aiming to furnish a theoretical framework for forest resource stewardship, eco-tourism strategizing, and national ecological security fortification.

## Materials and methods

### Overview of the study area

**Figure 1:**
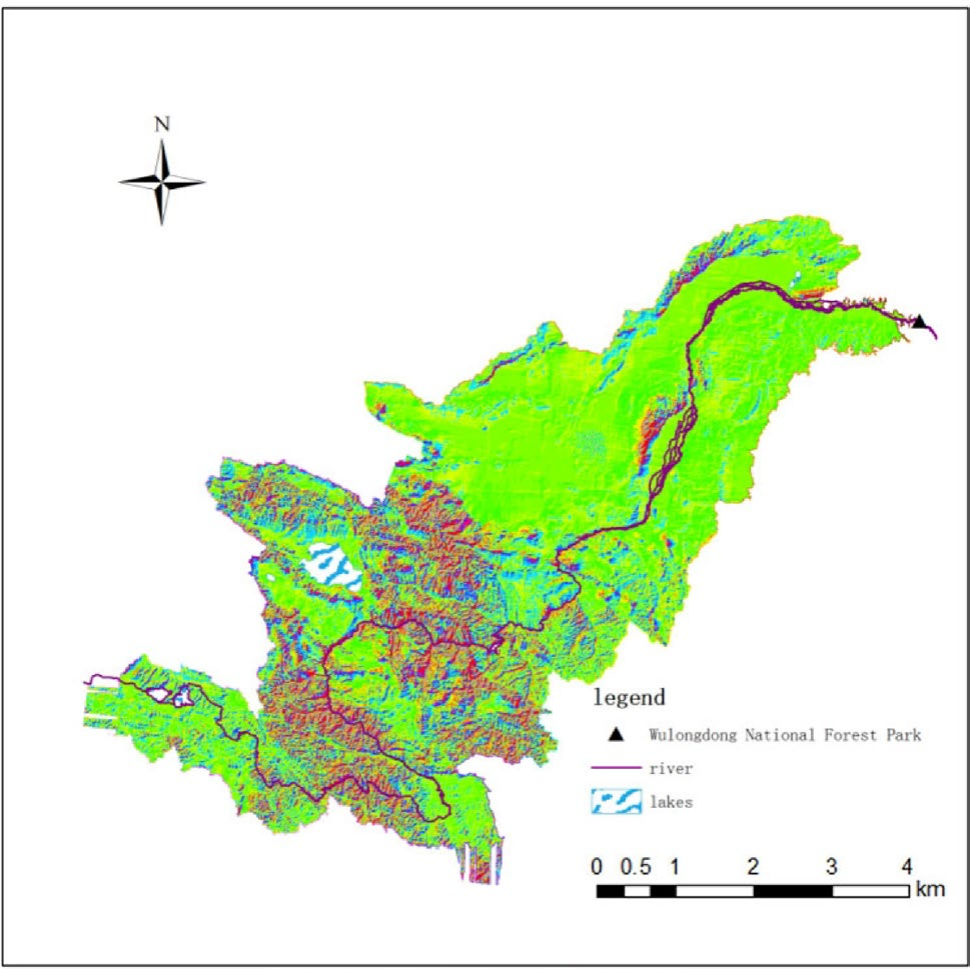
Geospatial map of Wulongdong National Forest Park. This picture was drawn using ArcGIS software. The version number: Arcgis 10.2. URL link: https://www.arcgis.com/index.html.

Wulongdong National Forest Park is located in Wulong Town, Linzhou City, at the junction of Anyang Linzhou, Hebi Qixian and Xinxiang Weihui counties (cities). The scenic spot is located at the eastern end of the Taihang Mountains. It is 7 km long from east to west and 6 km wide from north to south. It has a total area of 2525 hectares and includes four major scenic spots: Wulong Cave, Sidaogou, Jiguan Mountain and Lvtuogou (Fig. 1). The forest coverage rate in the park is as high as 93%, covering six major vegetation types and sixteen vegetation subtypes, with more than 300 species of plants. The main tree species include Japanese cedar, golden pine, five-needled pine and other precious species. They are affected by the ecological environment and Affected by the altitude, the negative oxygen ion index in the park is several times that of the urban area. It is known as the Henan version of "Zhangjiajie".

Wulongdong Forest National Park belongs to the South Taihang Mountains. The South Taihang Mountains are part of the Taihang Mountains, and their geological structures are mainly composed of the ancient Shanxi-Shaanxi Massif, the Western Henan Massif and the Henan Massif^19^. The bedrock in the South Taihang area mainly includes gneiss, granite, granulite, quartzite, etc. Rocks are the main material basis for soil formation, and mineral weathering provides rich mineral elements to the soil^20,21^. The content of major and trace elements in soil and its variation pattern on the profile are directly related to factors such as bedrock formation, soil texture, soil organic matter, and plant root distribution. The type and nature of bedrock directly affects the properties of the soil^22,23^. Different types of bedrock will produce different soil particle compositions during the weathering process, such as clay, loam, sand, etc. The mineral composition of rocks also affects soil chemistry^24,25^.

The rock types in Wulongdong Forest National Park mainly include quartzite and granite. These rocks are widely distributed in Wulongdong Forest National Park. The characteristics and patterns of rocks in Wulongdong Forest National Park are closely related to the geological history and tectonic background of the Taihang Mountains^26^. Henceforth, investigations conducted within analogous geological contexts can afford a profound comprehension of the intricate micro- and macro-properties of soil. This encompasses facets such as particle dispersion, organic matter composition, mineral content, moisture retention capacity, and beyond. Consequently, this research primarily delves into the geochemical attributes of soil heavy metal elements within the study region, thereby elucidating the content and spatial distribution characteristics of heavy metals within the forest soil of Wulongdong National Forest Park under prevailing environmental conditions.

### Sample collection and processing

This investigation and research employed a stratified-staggered-non-equilibrium approach to deploy sampling locations and methodically gather surface soil samples within the study region. The so-called stratification means that the study area is divided into sampling units of different levels according to the area size, and the sampling units of the higher level are integrated with the sampling units of the lower level; the so-called staggered-unbalanced means that the sampling units of the higher level are The units are not uniformly divided into the same number of low-level sampling units, but one or several are randomly selected from them to continue the division of the next-level sampling units^27,28^.

**Figure 2:**
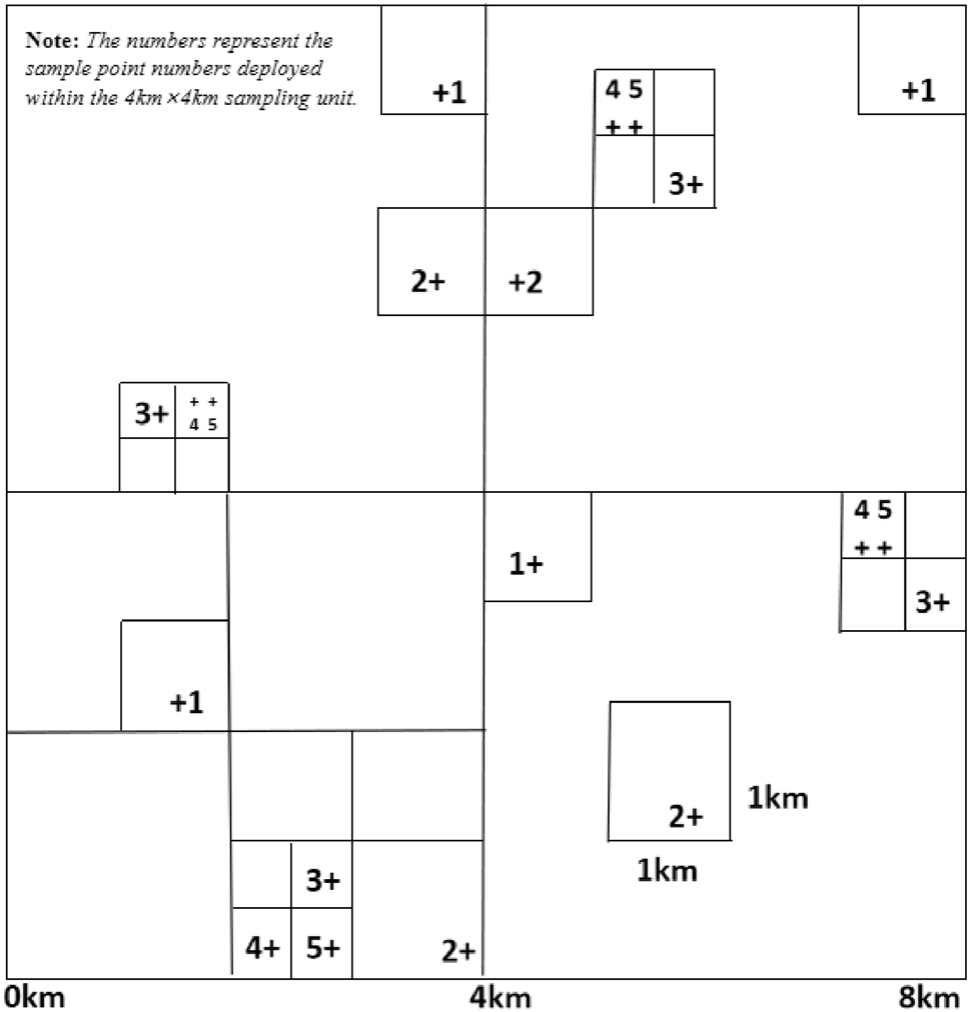
Layered staggered non-equilibrium mode sampling point deployment diagram.

The sample collection time is between November 2022 and May 2023, with 300 sampling points set up and a sampling depth of 20 cm. All sampling points are located within the scope of Wulongdong National Forest Park. During the sampling process, the sampling locations and surrounding environment were observed and recorded, and GPS was used for coordinate positioning. The distribution of sampling points is shown in Fig. 2.

The specific operation is as follows: Select 3 altitude gradients (900, 1000, 1069 m) on the shady slope and sunny slope of Wulongdong National Forest Park respectively, and select a sampling density 8 × 8 km grid as the top-level cell, which contains multiple lower levels. The nesting levels, such as 4 × 4 km, 2 × 2 km, 1 × 1 km, 500 × 500 m, 250 × 250 m, are randomly selected as the target cells (Fig. 2). According to the area of the study area, 30 top-level cells are deployed. In the case of affecting the layout of each level, each highest-level sampling cell contains 10 survey samples. The precise positioning of sampling points within each sampling cell can be meticulously adjusted manually based on soil characteristics, land classifications, and other relevant factors to ensure statistical accuracy across various categorizations. Once the requisite data statistical criteria are satisfied, this approach ensures meticulousness in the analysis.

**Figure 3:**
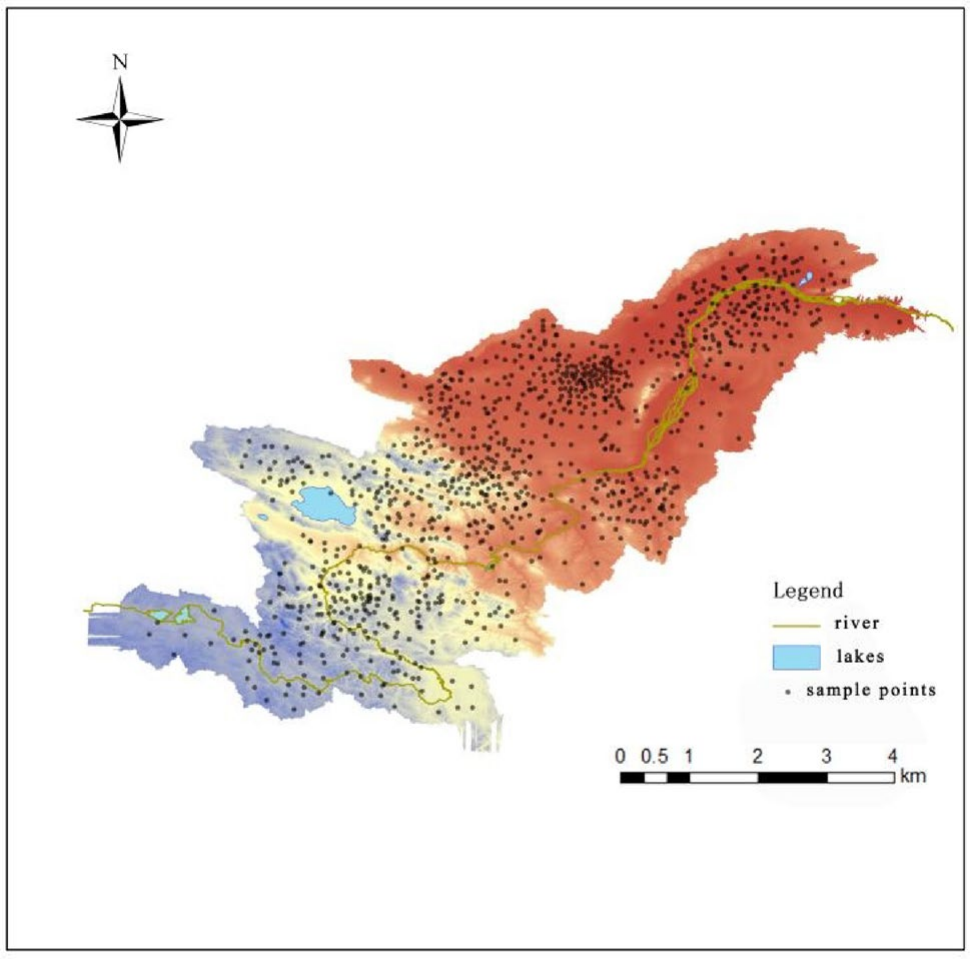
Distribution of sample points in Wulongdong National Forest Park. This picture was drawn using ArcGIS software. The version number: Arcgis 10.2. URL link: https://www.arcgis.com/index.html.

On the arranged sampling points, with the GPS positioning point as the center, 3–5 sampling points are determined 20–50 m in all directions, and equal parts are combined into a mixed sample, and 300 soil samples at a depth of 0–20 cm below the surface are collected. Building upon this foundation, soil layers spanning from 20 to 100 cm in depth were gathered as specimens for comparative analysis. ArcGIS 10.2 software was used to generate the sampling point distribution map (Fig. 3). When sampling, take samples in layers from bottom to top on the profile with the sampling depth marked, and collect about 200 g of samples from each layer. The collected soil samples were sealed in labeled polyethylene plastic bags and brought back to the laboratory. They were naturally air-dried at room temperature. Stones, plant residues and other debris were removed, and then ground and passed through a nylon mesh with a pore size of 0.147 mm. Samples were meticulously screened for further testing.

### Selection and determination of heavy metal elements

Metals possessing a specific gravity surpassing 5 are categorically denominated as heavy metals^29^ (Usually refers to those metals with a density greater than 4.5 g/cm^3^), such as Fe, Zn, Cd, Hg, Ni, Co, etc. In addition, the toxic effects and some properties of the metalloids Se and As are similar to those of heavy metals. Although they are not heavy metal elements, they are heavy metal pollutants^30^. There are many types of heavy metal elements that can cause pollution. In this study, the metal elements selected for determination in accordance with the long-term observation task indicators of forest soil in the "Terrestrial Ecosystem Soil Observation Specifications"^31^ include: chromium (Cr), nickel (Ni), lead (Pb), arsenic (As), cadmium (Cd), mercury (Hg), and selenium (Se).

### Sample determination method

According to the heavy metal element analysis method in the Soil Observation Specification for Terrestrial Ecosystems, the content of Hg, Se, and As was determined by 1 + 1 aqua regia digestion atomic fluorescence spectroscopy. The content of cadmium (Cd) was determined through digestion utilizing a combination of hydrochloric acid, nitric acid, hydrofluoric acid, and perchloric acid. The assessment of chromium (Cr) and nickel (Ni) content was conducted via digestion employing a combination of hydrochloric acid, nitric acid, hydrofluoric acid, and perchloric acid, followed by flame atomic absorption spectrophotometry.

### Evaluation method

#### Single-factor pollution index

Single-factor pollution index(Pi)The standard limit value of the background value of a certain pollutant in the soil is used as an evaluation index to measure the cumulative pollution degree of pollutants. It is a method of heavy metal pollution evaluation commonly used in China at present^32,33^. The calculation formula is:

$$ P_{i} = C_{i} /Cb_{i} $$

P_i_ is the pollution index of heavy metal element i in soil, C_i_ is the measured content of heavy metal element i, and C_b_ is the soil pollution risk screening value of agricultural land for heavy metal element i (GB15618-2018) (Note: GB15618-2018 is part of the Chinese national standard "Farmland Soil Environmental Quality Standard", which stipulates the limit values of various pollutants in farmland soil to assess the environmental quality of the soil.). When P_i_ ≤ 1, it means no pollution; when 1 < P_i_ ≤ 2, it means light pollution; when 2 < P_i_ ≤ 3, it means moderate pollution; when P_i_ > 3, it means heavy pollution.

#### Nemerow index method

Nemerow index method is developed from the single-factor pollution index method, which is one of the most commonly used methods to calculate the comprehensive pollution index at home and abroad. The impact of high-concentration pollutants on soil environmental quality^34,35^. A method of evaluation that comprehensively integrates multiple single-factor indices can provide a holistic depiction of the degree of pollution by various contaminants in soil (Table 1), and the specific calculation formula is:

$$ P_{n} = \sqrt {\frac{{\left( {P_{{_{imax} }}^{2} + P_{{_{{i{\text{average}}}} }}^{2} } \right)}}{2}} $$

**Table 1:** Evaluation grade of soil pollution by Nemerow comprehensive pollution index method.

Grade	P_n_	Pollution level	Pollution statement
I	P_n_ ≤ 0.7	Clean	Pollution-free (clean)
II	0.7 < P_n_ ≤ 1.0	Still clean (Warning line)	Not yet polluted (still clean)
III	1.0 < P_n_ ≤ 2.0	Light pollution	Soil is slightly polluted
IV	2.0 < P_n_ ≤ 3.0	Moderately polluted	The soil is moderately polluted
V	P_n_ > 3.0	Heavy pollution	The soil is polluted quite seriously

P_imax_: The maximum single-factor pollution index of each heavy metal element, P_iaverage_: The average value of the single factor pollution index of each heavy metal element, P_n_: Soil Nemerow Comprehensive Pollution Index.

#### Pollution load index method

The pollution load index method was proposed by Tomlinson, a British researcher on the classification of heavy metal pollution levels^36,37^. This method is composed of a variety of heavy metals contained in the evaluation area. The degree of heavy metal pollution at the sampling point can also be used to evaluate the comprehensive soil pollution status in a certain area^38,39^. Calculated as follows:

$$ P = \sqrt[{{}^{{\text{m}}}}]{{C_{f1} C_{f2} C_{f3} ......C_{fm} }},\,C_{fi} = \frac{{C_{i} }}{{C_{mi} }} $$

P is the pollution load index at a certain point; C_fi_ is the pollution factor of heavy metals. C_i_ is the measured value of heavy metal i; C_mi_ is the reference value of heavy metal i; m is the number of heavy metals involved in the evaluation.

The formula for calculating the pollution load index of a certain area is:

$$ P_{\alpha } = \sqrt[{{}^{n}}]{{P_{1} P_{2} P_{3} \cdots P_{n} }} $$

P_a_ is the pollution load index of a certain area, and n is the number of sampling points in this area. If P_a_ < 1, the area has no pollution; 1 ≤ P_a_ < 2, the area is lightly polluted; 2 ≤ P_a_ < 3, the area is moderately polluted; when P_a_ ≥ 3, the area is heavily polluted.

## Results and analysis

### Analysis of heavy metal elements in the forest soil of Wulongdong National Forest Park

Descriptive statistical analysis was conducted on the soil samples obtained from the 0–20 cm soil stratum within the study area. This analysis yielded the minimum, maximum, mean, standard deviation, and coefficient of variation of the concentrations of seven heavy metal elements. The results are shown in Table 1.

As delineated in Table 2, the mean levels of heavy metal elements Cr, Se, As, and Pb within the 0–20 cm soil stratum of Wulongdong National Forest Park reside beneath the national soil baseline and the soil benchmarks of Tibet. Yet, the mean concentrations of Cd and Hg surpass not only the national soil standards but also those of Henan province. Specifically, they exhibit ratios of 2.2 and 2.92 times the national soil benchmarks^40^, and 2.75 and 9.5 times the soil benchmarks of Henan^41–43^.

**Table 2:** Statistical analysis of heavy metal content in the 0–20 cm soil layer of Wulongdong National Forest Park.

Heavy metal elements	Average value /mg·kg^−1^	Minimum value/mg·kg^−1^	Maximum value/mg·kg^−1^	Standard deviation/mg·kg^−1^	Coefficient of variation/CV	Background value in Chian/mg·kg^−1^	Background value in Henan/mg·kg^−1^
Cr	29.80	6.00	81.00	15.59	52.33	61.00	76.60
Ni	27.63	5.00	153.00	18.68	67.60	26.90	32.10
Cd	0.22	0.10	0.54	0.11	51.72	0.10	0.08
Se	0.11	0.02	0.40	0.08	75.45	0.29	0.16
As	10.48	0.32	29.50	5.73	54.69	11.20	19.70
Hg	0.19	0.01	0.88	0.19	101.13	0.065	0.02
Pb	24.25	1.83	66.13	12.61	51.98	26.00	29.10

The coefficient of variation (CV) reflects the degree of dispersion of data among sample points. CV < 20% is low variation, 20% ≤ CV < 50% is medium variation, 50% ≤ CV < 100% is high variation, and CV ≥ 100% is extreme mutation. The coefficients of variation of the seven heavy metal elements were all greater than 50%, among which Hg showed extreme variation, and the remaining six belonged to high variation.

### Spatial distribution characteristics of heavy metals in forest soil in Wulongdong National Forest Park

#### Heavy metal content in soil at different altitudes

The concentrations of seven heavy metal elements in soils at varying elevations are detailed in Table 3. On sunlit slopes, there is a discernible decline in the levels of As, Cd, and Hg with increasing altitude, while Se levels exhibit an ascent. Conversely, the correlation between Cr, Pb, and Ni concentrations and altitude remains indistinct. Specifically, the levels of Cr, Se, and As diminish with altitude, while those of Pb and Hg escalate. Meanwhile, the levels of Ni and Cd demonstrate no clear association with altitude.

**Table 3:** Heavy metal content in forest soil at different altitudes in Wulongdong National Forest Park (mg/kg).

Slope aspect	Elevation/m	Cr	Ni	Pb	As	Cd	Hg	Se
Sunny slope	900	27.87	28.30	30.22	12.43	0.23	0.27	0.09
	1000	26.90	39.70	21.77	10.62	0.22	0.22	0.14
	1069	31.07	23.90	27.43	5.85	0.21	0.10	0.17
Slady slope	900	36.40	13.50	12.89	14.19	0.21	0.08	0.22
	1000	35.42	30.96	29.51	12.68	0.24	0.14	0.08
	1069	32.88	28.15	34.52	10.40	0.22	0.17	0.08

#### Vertical distribution characteristics of soil heavy metals

**Figure 4:**
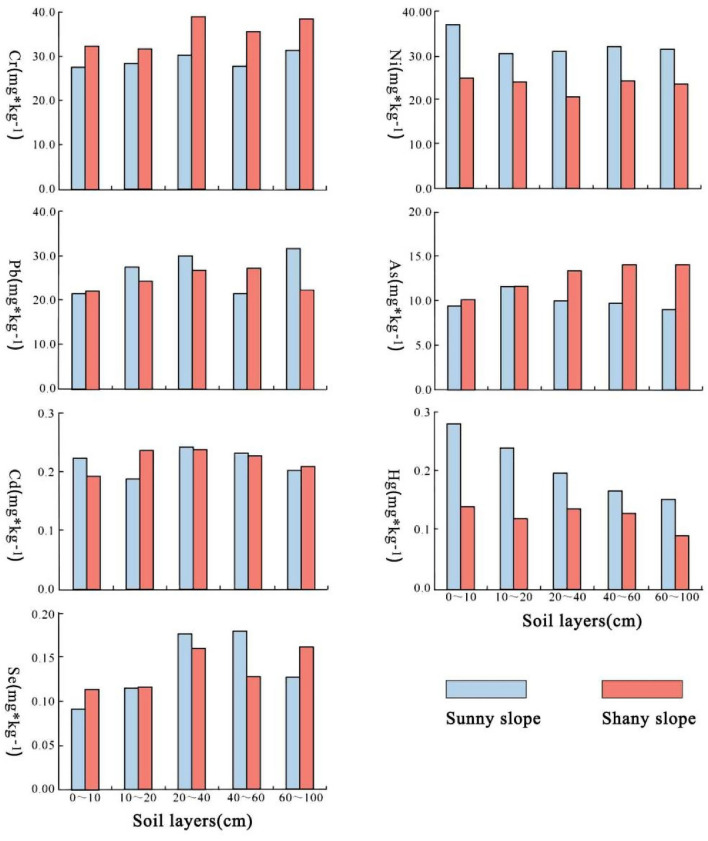
Distribution of heavy metal content in different soil layers on shady and sunny slopes in Wulongdong National Forest Park.

Observation of Fig. 4 discerns a progressive augmentation in the concentration of element As concomitant with the deepening of soil layers upon shaded slopes. Conversely, a roportional diminution in the concentration of element Hg is discerned with the increasing profundity of soil layers on sunlit slopes. The concentrations of other heavy metal elements exhibit no discernible regularities across soil.

At the same soil depth, the concentrations of Cr and As metals were generally lower on the sunny slope compared to the shady slope, while the contents of Pb, Ni, and Hg were overall higher on the sunny slope than on the shady slope. For Cd, the metal content at soil depths of 0–10 cm, 20–40 cm, and 40–60 cm was higher on the sunny slope than on the shady slope, whereas at depths of 10–20 cm and 60–100 cm, it was lower on the sunny slope compared to the shady slope. As for Se, the metal content at depths of 0–10 cm, 10–20 cm, and 60–100 cm was lower on the sunny slope than on the shady slope, whereas at 20–40 cm and 40–60 cm depths, it was higher on the sunny slope than on the shady slope.

### Evaluation and analysis of forest soil heavy metal pollution in Wulongdong National Forest Park

#### Evaluation of the single factor index method

With reference to the national background value of soil elements^44–46^, a comprehensive evaluation was made of the heavy metal pollution in the 0–20 cm soil layer of Wulongdong National Forest Park. It can be seen from Table 4 that on sunny slopes, the order of the average value of the soil heavy metal single-factor pollution index Pi is Hg > Cd > Ni > Pb > As > Cr > Se, where Hg is heavily polluted, Cd is moderately polluted, and Ni is lightly polluted. Degrees of pollution, Cr, Se, As, and Pb are non-pollution states.

**Table 4:** Comprehensive pollution index of heavy metals in the 0–20 cm soil layer of Wulongdong National Forest Park.

Slope aspect	Elevation/m	P_Cr_	P_Ni_	P_Pb_	P_As_	P_Cd_	P_Hg_	P_Se_	(P_i_)ave	(P_i_)_max_	P_complex_
Sunny slope	900	0.40	1.01	1.26	1.16	2.30	5.50	0.30	1.70	5.50	4.07
	1000	0.44	1.66	0.96	1.03	2.19	3.94	0.44	1.52	3.94	2.99
	1069	0.58	0.97	0.65	0.54	1.65	2.29	0.34	1.00	2.29	1.77
	Average value	0.47	1.21	0.96	0.91	2.05	3.91	0.36	1.41	3.91	2.94
Slady slope	900	0.67	0.43	0.46	1.12	2.08	1.28	0.78	0.97	2.08	1.63
	1000	0.53	1.13	1.09	1.07	2.17	2.17	0.23	1.20	2.17	1.75
	1069	0.37	1.16	1.15	0.73	2.17	2.62	0.18	1.19	2.62	2.04
	Average value	0.52	0.91	0.90	0.97	2.14	2.02	0.39	1.12	2.14	1.71

On the shady slopes, the order of single-factor pollution index Pi of heavy metals in soil was Cd > Hg > As > Ni > Pb > Cr > Se, in which Cd and Hg were moderately polluted and the other five heavy metal elements were all non-polluted.

Furthermore, within the soils of sunlit slopes, the extent of heavy metal pollution stemming from elements such as As, Pb, Cd, and Hg exhibited a decline with ascending altitudes, while that of Cr demonstrated an incline. Conversely, within the soils of shaded slopes, the pollution levels of Cr and Se, alongside the extent of As contamination, diminished with increasing altitudes. In contrast, the pollution levels of Cd, Hg, Ni, and Pb experienced a rise in tandem with altitude elevation.

#### Nemerow index method evaluation

Table 4 delineates the comprehensive pollution index, revealing that within Wulongdong National Forest Park, the 0–20 cm soil stratum is substantially more contaminated by heavy metals on sunlit slopes in comparison to shaded slopes. The comprehensive pollution degree of heavy metals on sunny slopes decreased with the increase in altitude. The soil at an altitude of 900 m was heavily polluted, the soil at an altitude of 1000 m was moderately polluted, and the soil at an altitude of 1069 m was slightly polluted.The degree of pollution exhibited a positive correlation with altitude. Soil at altitudes of 900 and 1000 m exhibited slight pollution, while soil at an altitude of 1069 m showed a moderate level of contamination.

#### Evaluation of pollution load index method

The Pollution Load Index methodology was utilized to evaluate the status of heavy metal pollution within the 0–20 cm soil stratum of Wulongdong National Forest Park. The findings, as delineated in Table 5, elucidate that the soil in the sunlit slope region at an elevation of 4300 m remains untainted by heavy metal pollutants. Conversely, the soil in region m exhibits a subtle contamination from heavy metals. In general, the forest soil on sunlit slopes exhibited a minor degree of heavy metal contamination, whereas the soil on shaded slopes remained devoid of heavy metal pollution.

**Table 5:** Heavy metal pollution load index of 0–20 cm soil layer in Wulongdong National Forest Park.

Slope aspect	Elevation/m	P
Sunny slope	900	1.12
	1000	1.15
	1069	0.82
	Average value	1.02
Slady slope	900	0.85
	1000	0.96
	1069	0.87
	Average value	0.89

## Discussion

The deleterious effects of heavy metals on forest soil are enduring, pernicious, and to a certain extent, irreversible. Consequently, the investigation into the content and spatial distribution characteristics of heavy metals in soil has long been esteemed by scholars both domestically and internationally As the population continues to burgeon and rapid economic advancement persists, human endeavors have profoundly altered the natural distribution of heavy metal elements. Research indicates that even remote virgin forests, far removed from urban centers, bear the burden of heavy metal pollution^47–49^.

This experiment investigated the concentrations of heavy metals in the soil across varying slope aspects, altitudinal gradients, and depths within Wulongdong National Forest Park. From the perspective of the comprehensive pollution index, the degree of heavy metal pollution in sunny slope soil decreased gradually with the increase in altitude, but in the shade The pollution degree of heavy metals in slope soil increased with elevation, and the pollution degree of a sunny slope was more serious than that of a shady slope. It is conceivable that the solar inclinations offer superior luminosity compared to their shaded counterparts owing to an earlier dissipation of ice and snow. Furthermore, at lower elevations, there is an increased incidence of biological activities. A multitude of biological processes exert a perceptible impact on the composition of soil constituents, consequently exacerbating pollution in direct correlation with the descent in altitude on sunlit slopes. Conversely, shaded inclines, perennially deprived of ample sunlight, grapple with prolonged ice and snow cover, impeding the vigor of biological activities. As altitude increases, biological activity diminishes^50–52^, leading to a more pronounced presence of heavy metal pollution on sunny slopes as compared to shady slopes.

Heavy metal contamination emanates not only from the natural erosion of rock formations but also from anthropogenic sources of pollution. We shall delve into the potential sources of contamination, encompassing industrial complexes, urban conglomerations, and other human activities that could influence heavy metal concentrations within the study area.

The Taihang Mountain Expressway and Taihui Expressway traverse the mountains, providing convenient access to the scenic region but also contributing to environmental degradation. These highways exhibit divergent effects on soil heavy metal content across the shaded and sunlit facets of Wulongdong Forest Park. Soil on the shaded side may be subject to deposition of traffic exhaust fumes and atmospheric pollutants, resulting in heightened heavy metal levels, whereas soil on the sunny side may be primarily influenced by solar irradiation and natural soil processes, thus maintaining relatively lower heavy metal content.

Similarly, heavy industrial enclaves like the Dingjiao Industrial Zone, Hejian Industrial Park, and Linzhou Chemical Plant situated in Linzhou City may exert varying impacts on soil heavy metal concentrations across the shaded and sunlit expanses of Wulongdong Forest Park. Soil on the shaded side could be directly impacted by industrial discharges, leading to elevated heavy metal levels, while soil on the sunny side may be more susceptible to degradation by sunlight and natural ventilation, thereby maintaining comparatively lower heavy metal content.

In this investigation, various pollution assessment methodologies exert profound influence on the evaluation outcomes. The findings derived from the Nemerow index method revealed a discernible presence of heavy metal contamination in the soil at sampling sites across both the shaded and sunlit slopes of Wulongdong National Forest Park. Conversely, the results obtained from the pollution load index method indicated that only the sampling points situated on the sunlit slopes exhibited mild heavy metal pollution. This disparity may stem from the pronounced excess of Cd and Hg content in the soil of both sunlit and shaded slopes. The Nemerow index method tends to magnify the impact of high-content factors or diminish the role of low-content factors to a certain extent, which could contribute to this observed divergence^53,54^.

## Conclusion

1. In the 0–20 cm soil layer of Wulongdong National Forest Park, among the seven heavy metal elements measured, except for Cr, Se, As, and Pb, the other three heavy metal elements have reached the pollution level, of which Ni is slightly Pollution, Cd and Hg are moderate or above pollution; in addition, the variation coefficients of the seven heavy metal elements are all higher than 50%, especially Hg is extremely variable, and the variation coefficient is greater than 100%.
2. The spatial distribution of soil heavy metal content in Wulongdong National Forest Park is affected by slope aspect, altitude, and soil depth, and the slope aspect has the greatest impact. The content of Cr and As in sunny slopes is lower than that in shady slopes, and the content of Ni, Pb, and Hg in sunny slopes is higher than that in shady slopes. On sunny slopes, the contents of As, Cd, and Hg decreased with increasing altitude, while the content of Se increased with increasing altitude; on shady slopes, the contents of Cr, Se, and As decreased with increasing altitude, while Pb and Hg content increased with altitude. The As content of heavy metal elements increased with the deepening of the soil layer on the shady slope, and the content of Hg decreased with the deepening of the soil layer on the sunny slope.
3. Through the comprehensive evaluation of heavy metal pollution in the 0–20 cm soil layer of Wulongdong National Forest Park, it was found that both sunny and shady slopes had different degrees of heavy metal pollution, and the pollution degree of sunny slopes was greater than that of shady slopes. Analyzing the single factor index of 7 heavy metal elements, the main cause of soil heavy metal pollution is the high single factor index of Cd and Hg, and using the pollution load index method to analyze the heavy metal pollution in the 0–20 cm soil layer of Wulongdong National Forest Park The evaluation results showed that the sunny slope forest soil was slightly polluted by heavy metals, and the shady slope had no heavy metal pollution.

## Deficiencies and prospects

This study designated Wulongdong National Forest Park as its primary research locus, collecting samples from 300 distinct sampling sites spanning varied altitudinal gradients across both shaded and sunlit slopes. While the scrutiny and assessment of seven heavy metal constituents were conducted meticulously, empirical surveys encountered limitations in sample selection owing to temporal, financial, and geographical constraints. These limitations resulted in a constrained sample scope, potentially falling short of fully encapsulating the entirety of soil conditions within the forest park.

The concentration of heavy metals in soil is intricately influenced by numerous factors, encompassing human activities, as well as natural processes. The intricate interplay of these elements poses challenges in accurately discerning the precise contribution of each factor to the soil's heavy metal content during analysis, thereby potentially impeding a profound understanding of the underlying causes of soil pollution.

In areas where soils exhibit heightened concentrations of heavy metals, a vigilant emphasis should be placed on mitigating pollution resulting from human and ancillary activities, thereby reducing the likelihood of burgeoning mild contamination. Nevertheless, given that urban forest parks are frequented by human activity, forthcoming research endeavors will delve into the influence of human factors on the structural integrity of forest soil. These endeavors aim to mitigate heavy metal pollution within Wulongdong National Forest Park while upholding the ecological equilibrium of the park. Additionally, the establishment of mathematical models to forecast future trends in soil heavy metal concentrations and assess environmental and human health risks will be pursued. Such endeavors are integral to crafting more effective protection strategies and decisions.

In conclusion, inadequacies persist in the scrutiny of soil heavy metal content and pollution assessment within Wulongdong National Forest Park, particularly concerning the realms of data collection and the multifaceted analysis of contributing factors. A comprehensive and profound elucidation of research findings is imperative, aimed at furnishing a scientific foundation for the sustainable evolution of protected areas.

## Supplementary Information

Supplementary Information.

## Data Availability

The datasets used and/or analysed during the current study available from the corresponding author on reasonable request.
