# Supplementary figures and images for: Spatial distribution characteristics and pollution evaluation of soil heavy metals in Wulongdong National Forest Park
Source: Sci Rep. 2024 Apr 17;14:8880. doi: 10.1038/s41598-024-58259-5 (PMC11525477; doi:10.1038/s41598-024-58259-5)

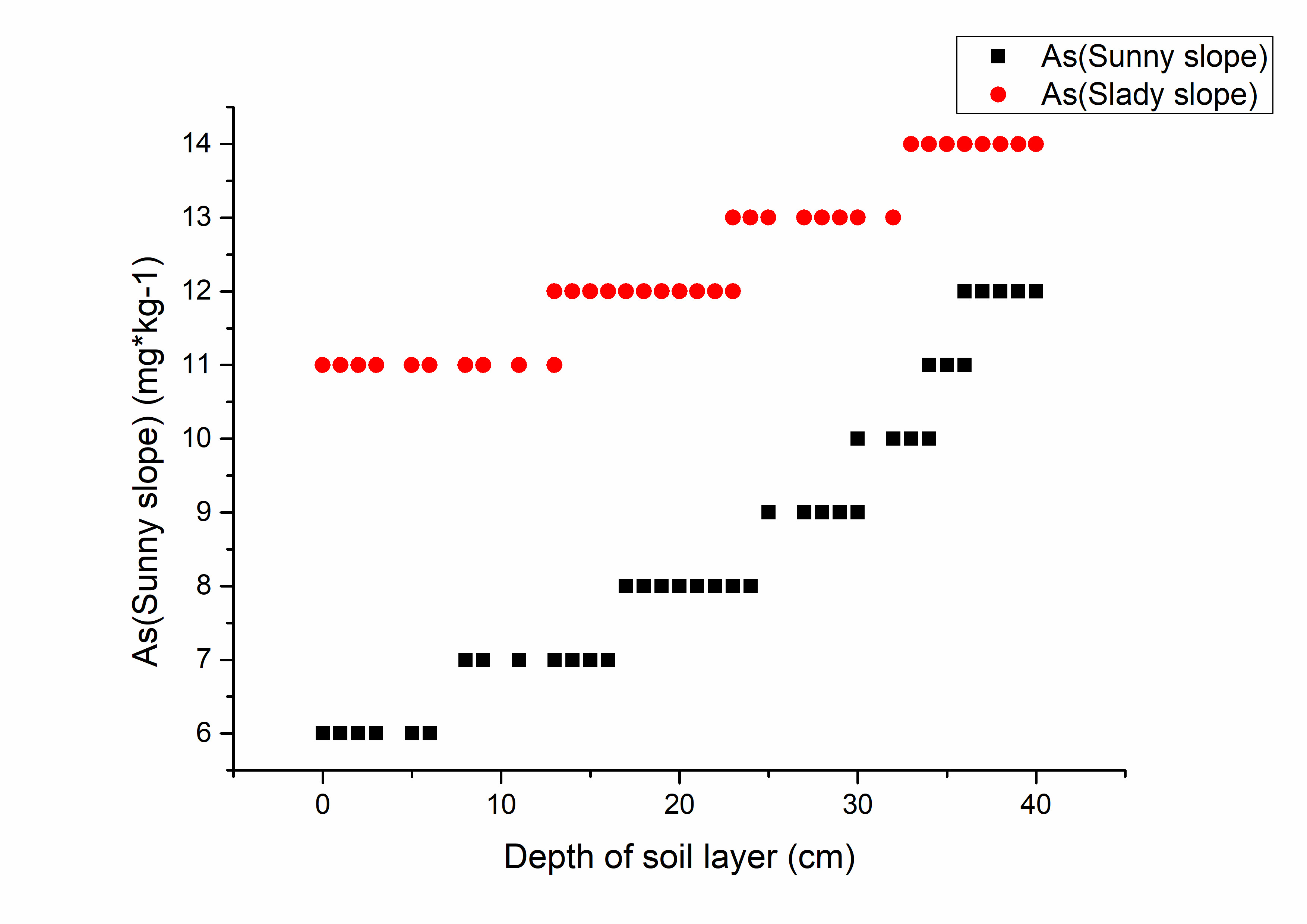

Supplement: Supplementary file 1 — Supplementary Information. [file 41598_2024_58259_MOESM1_ESM.zip › Raw data/data analysis/as.jpg]

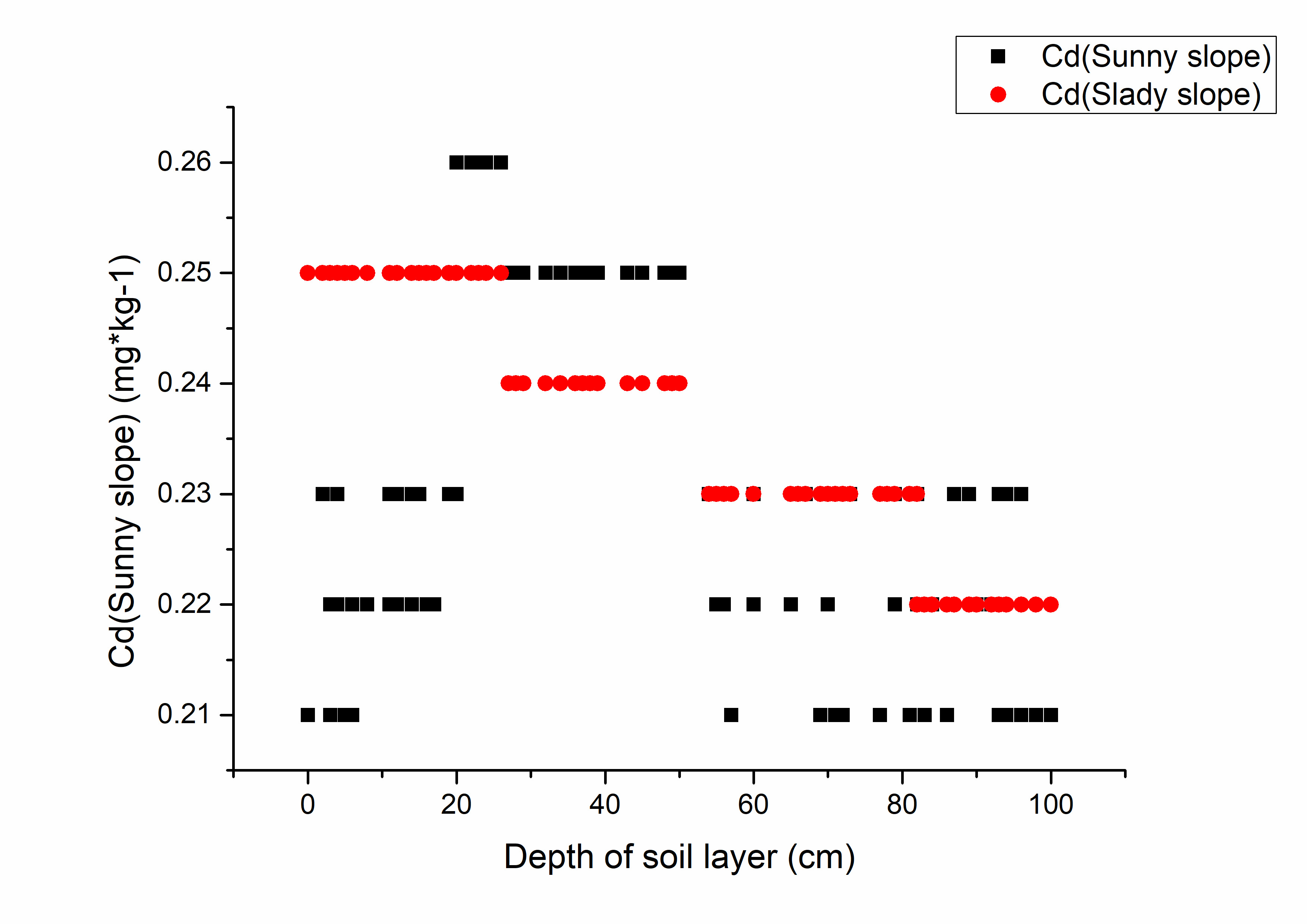

Supplement: Supplementary file 1 — Supplementary Information. [file 41598_2024_58259_MOESM1_ESM.zip › Raw data/data analysis/cd.jpg]

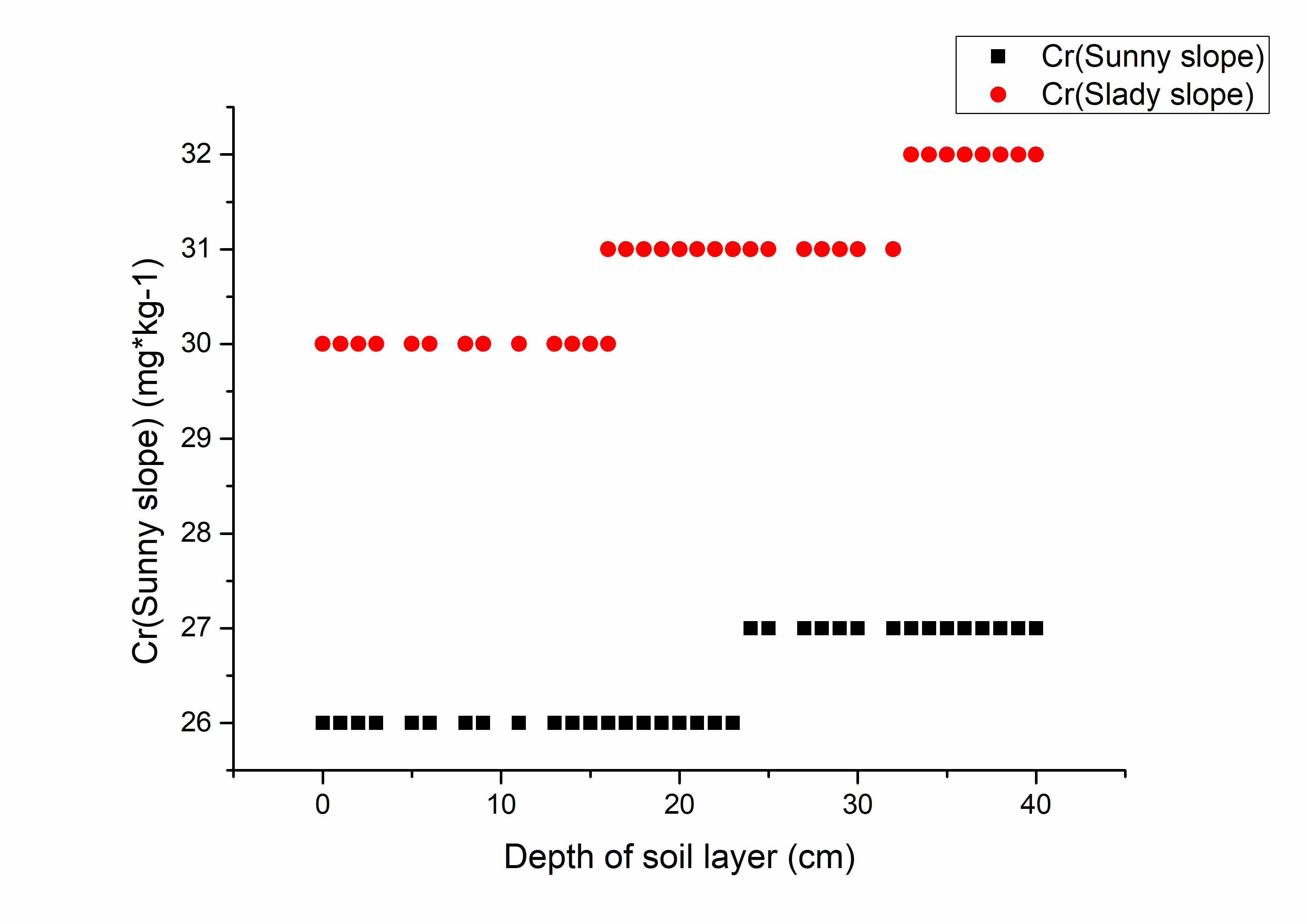

Supplement: Supplementary file 1 — Supplementary Information. [file 41598_2024_58259_MOESM1_ESM.zip › Raw data/data analysis/cr.jpg]

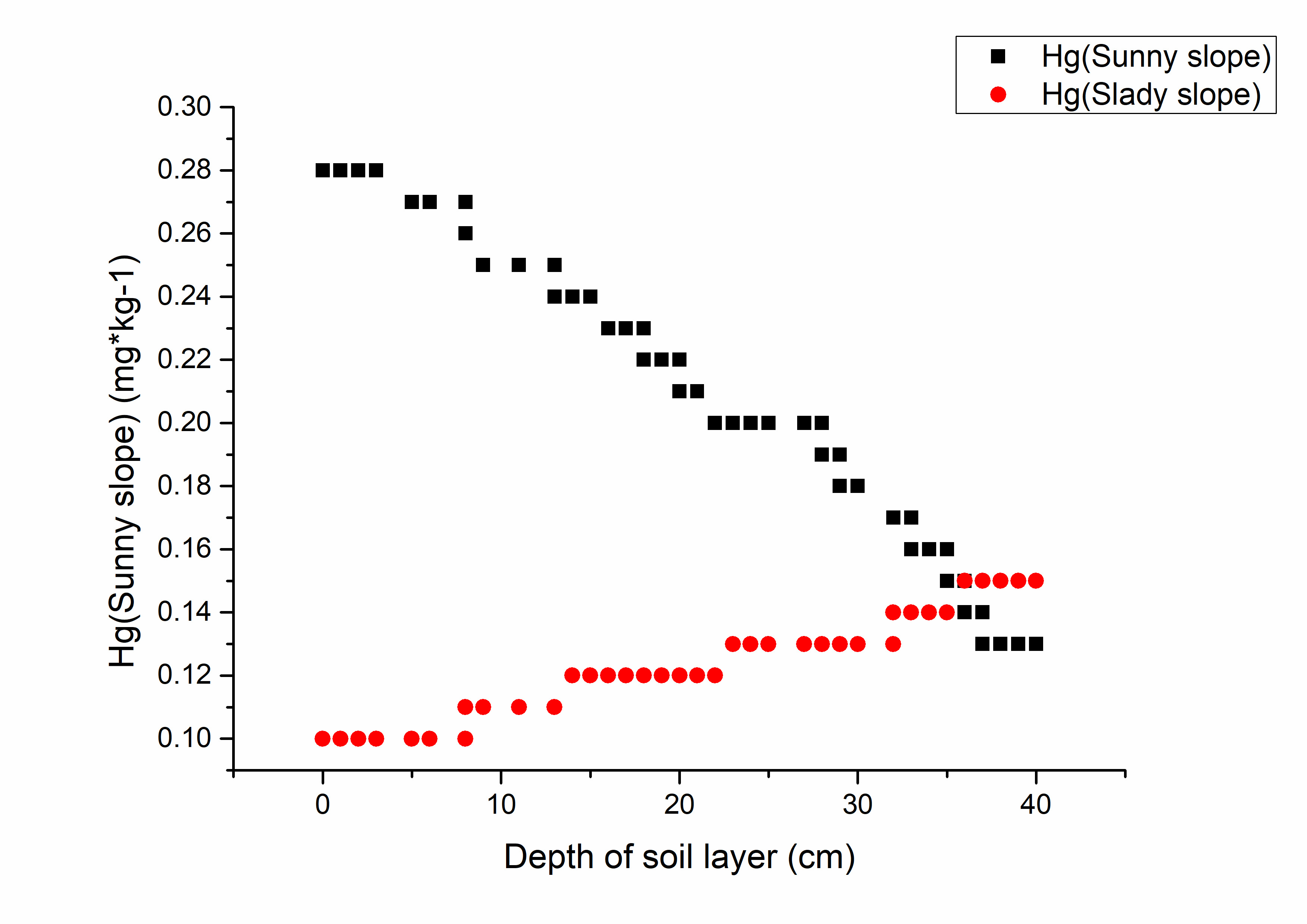

Supplement: Supplementary file 1 — Supplementary Information. [file 41598_2024_58259_MOESM1_ESM.zip › Raw data/data analysis/hg.jpg]

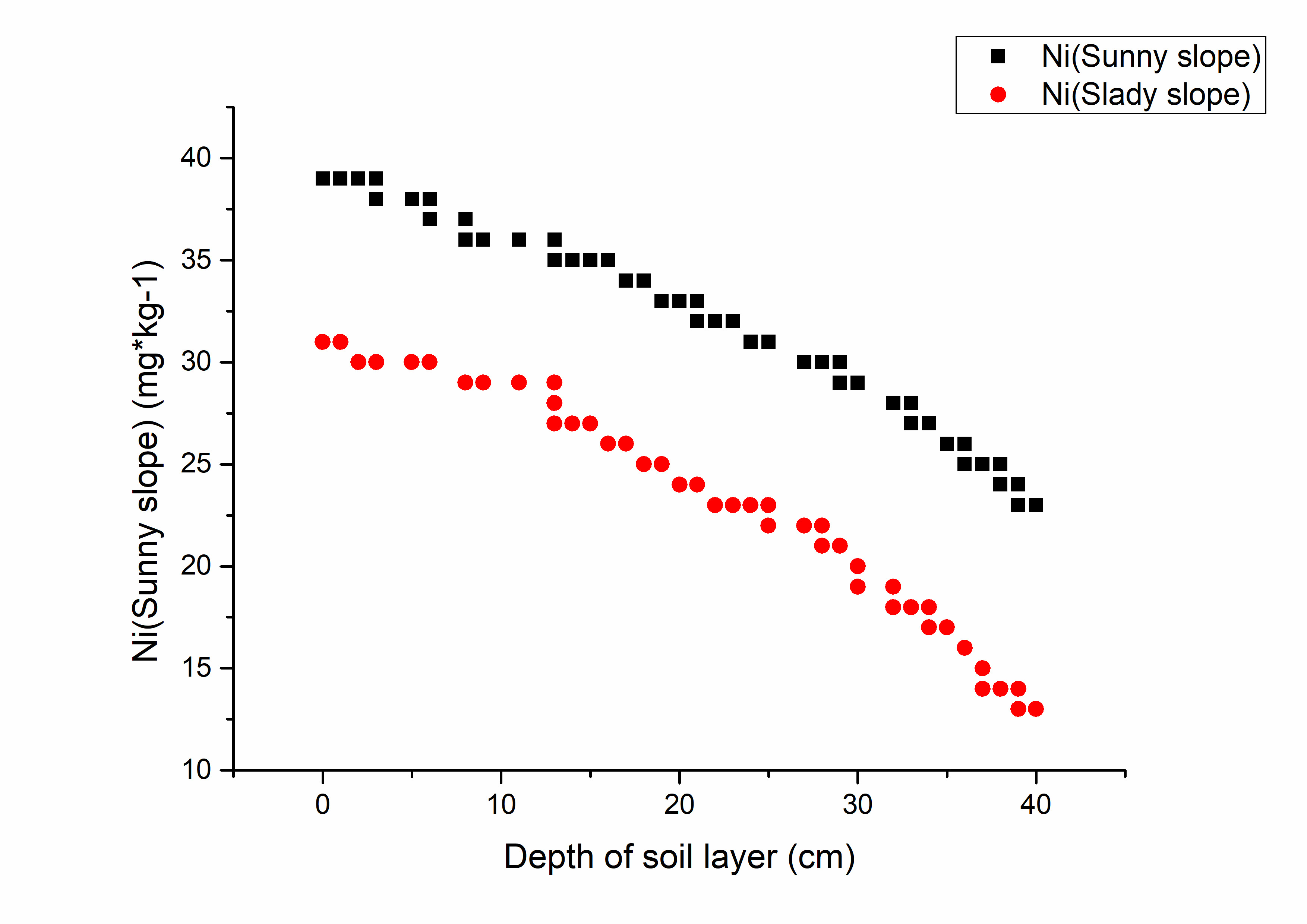

Supplement: Supplementary file 1 — Supplementary Information. [file 41598_2024_58259_MOESM1_ESM.zip › Raw data/data analysis/ni.jpg]

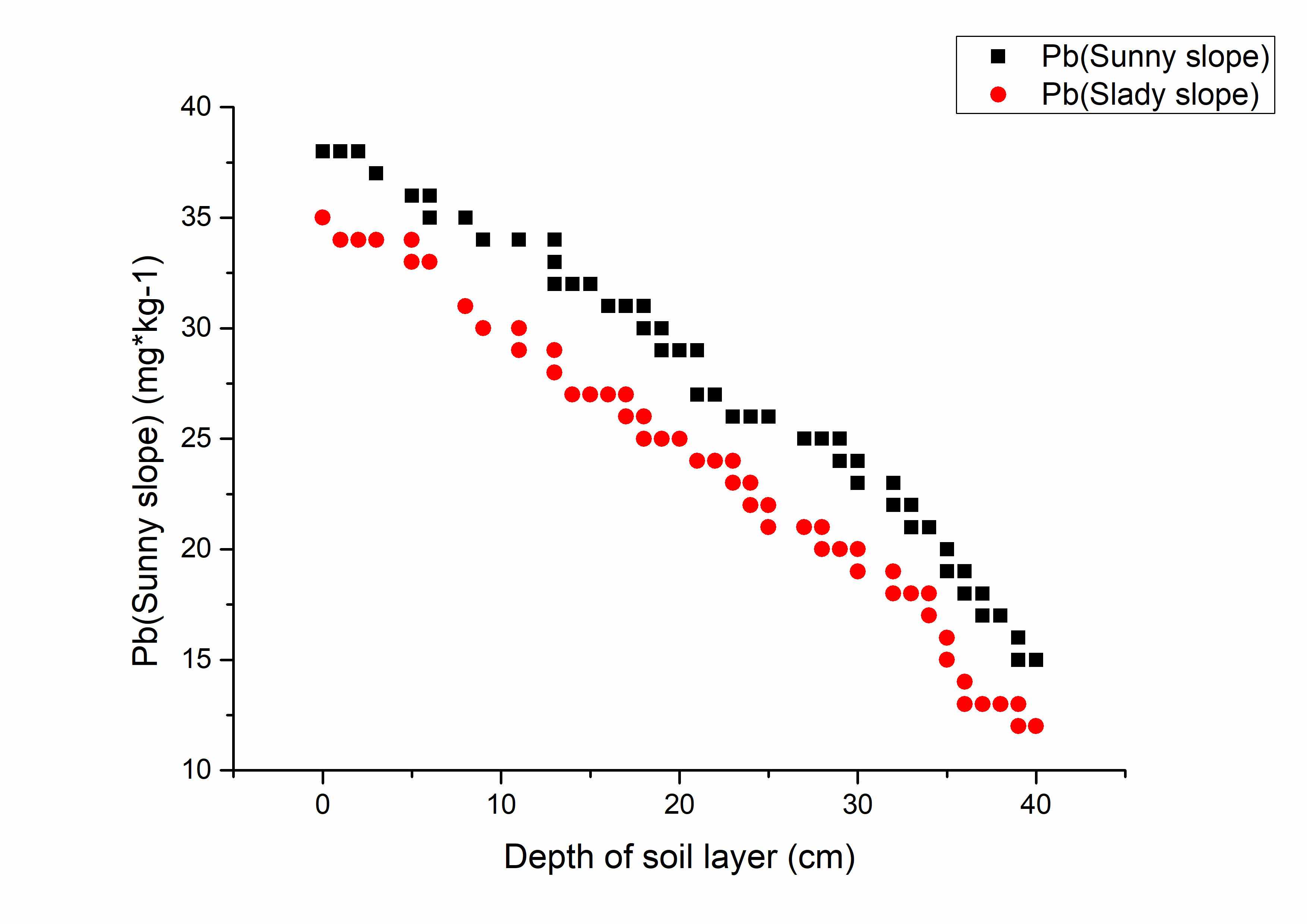

Supplement: Supplementary file 1 — Supplementary Information. [file 41598_2024_58259_MOESM1_ESM.zip › Raw data/data analysis/pb.jpg]

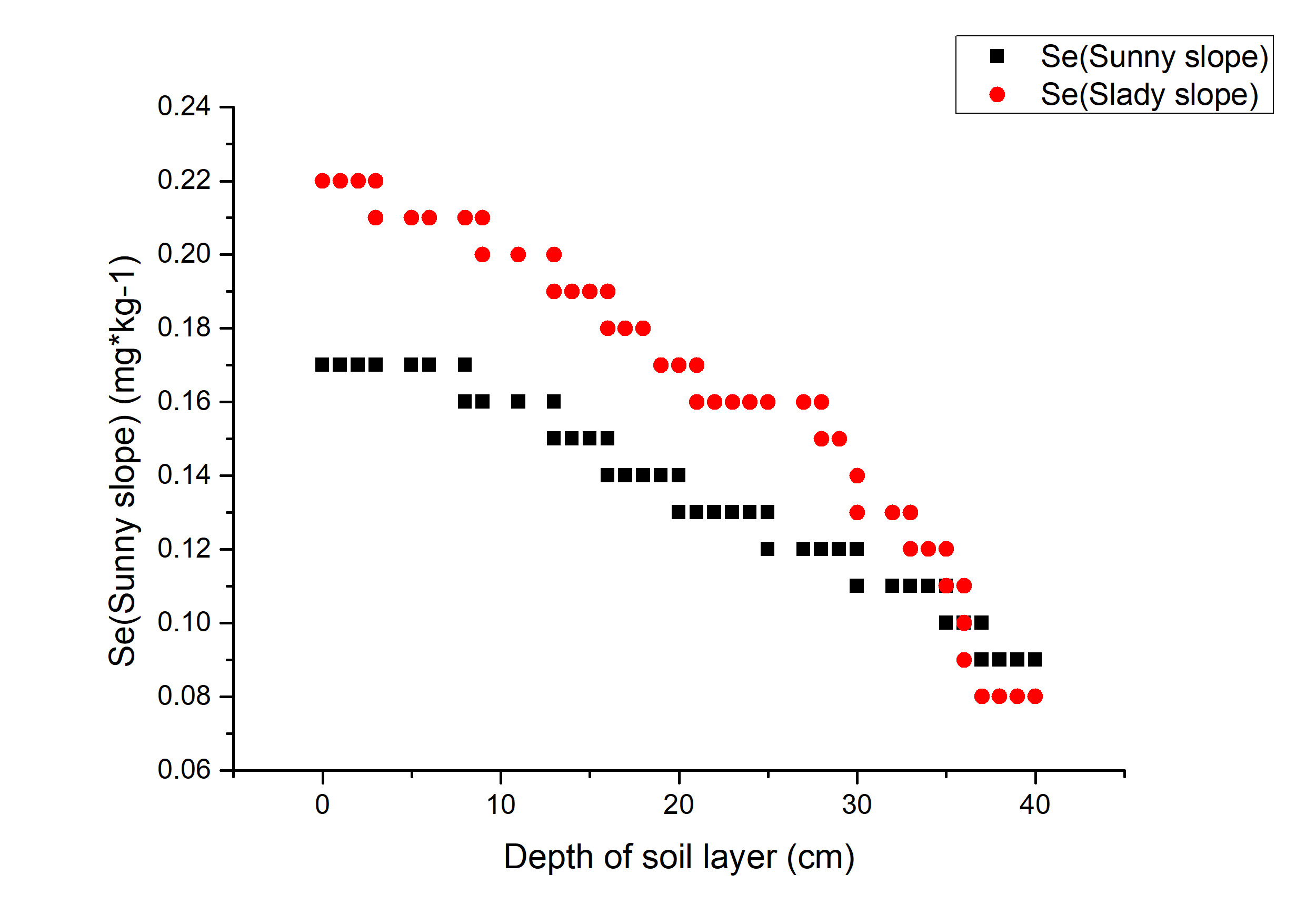

Supplement: Supplementary file 1 — Supplementary Information. [file 41598_2024_58259_MOESM1_ESM.zip › Raw data/data analysis/se.jpg]

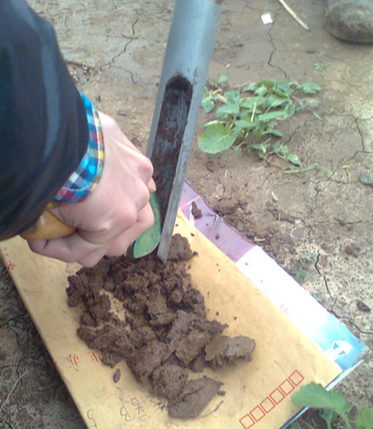

Supplement: Supplementary file 1 — Supplementary Information. [file 41598_2024_58259_MOESM1_ESM.zip › Raw data/Sample sampling site photos/1 (1).png]

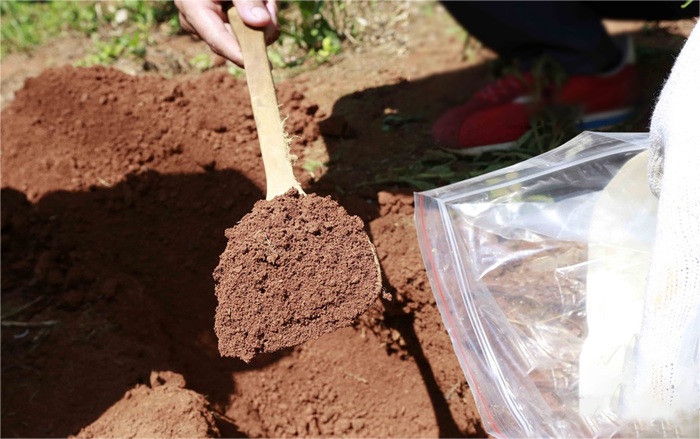

Supplement: Supplementary file 1 — Supplementary Information. [file 41598_2024_58259_MOESM1_ESM.zip › Raw data/Sample sampling site photos/1 (10).png]

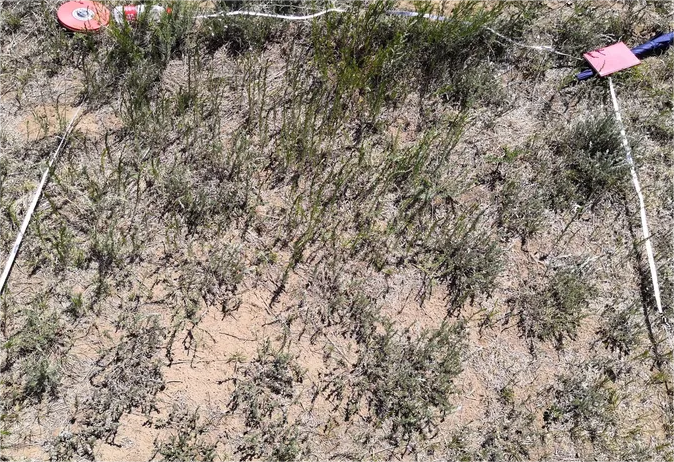

Supplement: Supplementary file 1 — Supplementary Information. [file 41598_2024_58259_MOESM1_ESM.zip › Raw data/Sample sampling site photos/1 (11).png]

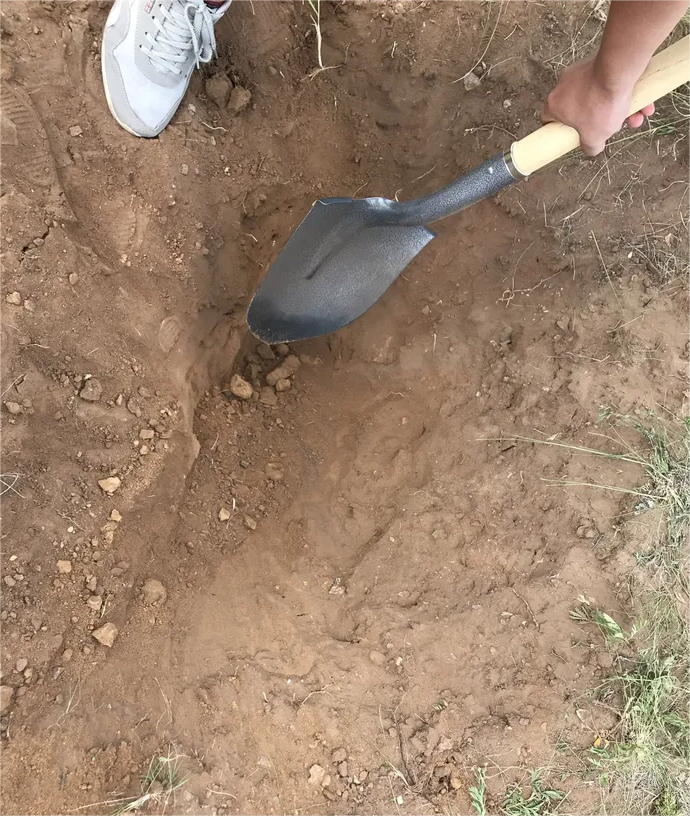

Supplement: Supplementary file 1 — Supplementary Information. [file 41598_2024_58259_MOESM1_ESM.zip › Raw data/Sample sampling site photos/1 (12).png]

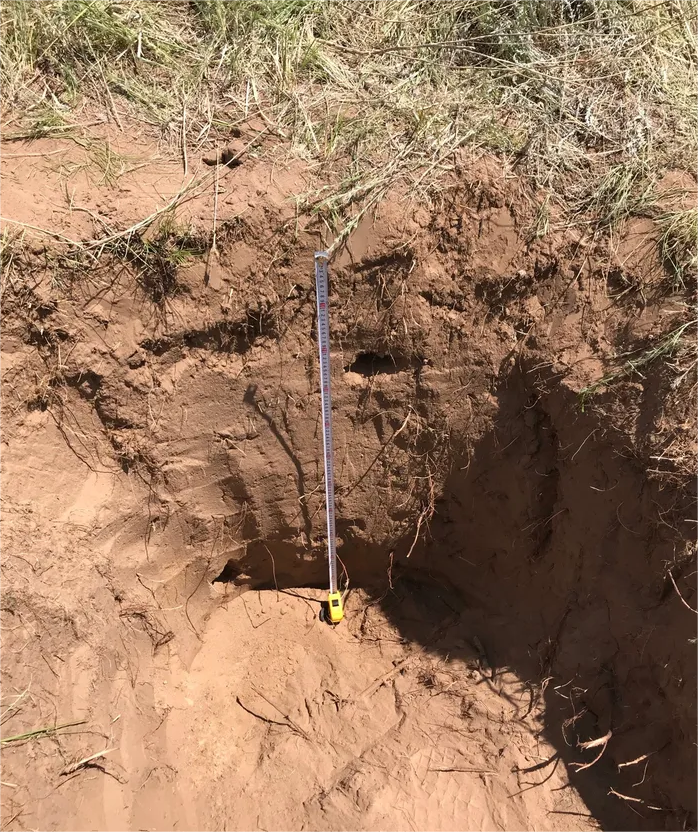

Supplement: Supplementary file 1 — Supplementary Information. [file 41598_2024_58259_MOESM1_ESM.zip › Raw data/Sample sampling site photos/1 (13).png]

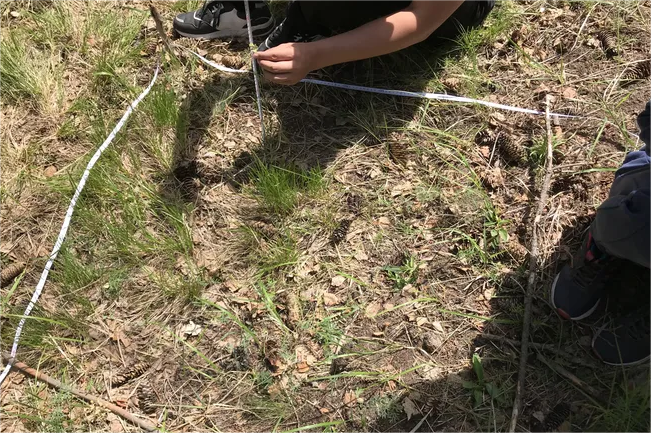

Supplement: Supplementary file 1 — Supplementary Information. [file 41598_2024_58259_MOESM1_ESM.zip › Raw data/Sample sampling site photos/1 (14).png]

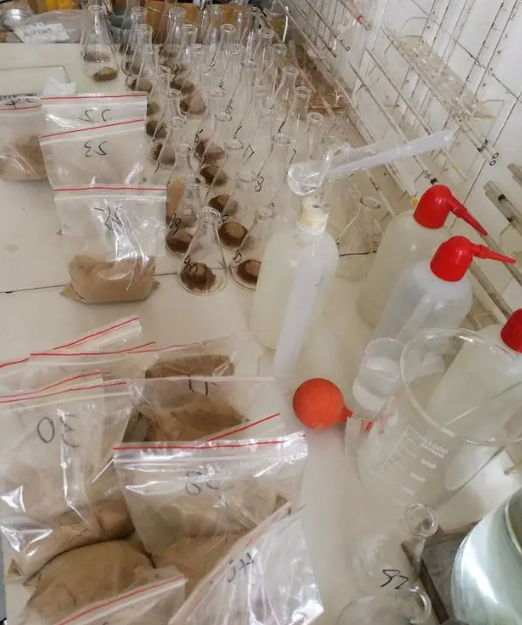

Supplement: Supplementary file 1 — Supplementary Information. [file 41598_2024_58259_MOESM1_ESM.zip › Raw data/Sample sampling site photos/1 (4).png]

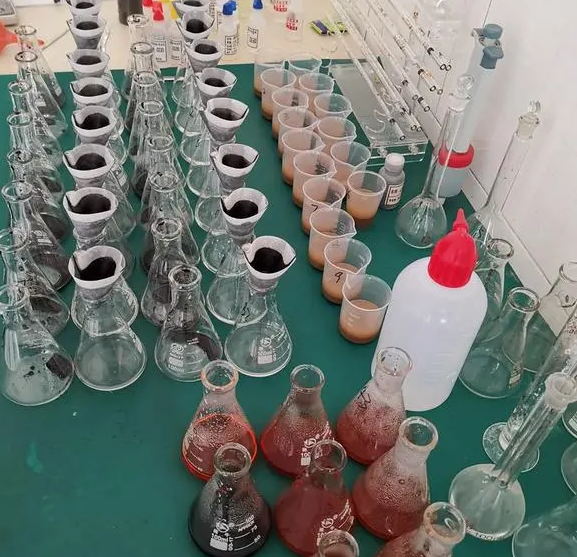

Supplement: Supplementary file 1 — Supplementary Information. [file 41598_2024_58259_MOESM1_ESM.zip › Raw data/Sample sampling site photos/1 (5).png]

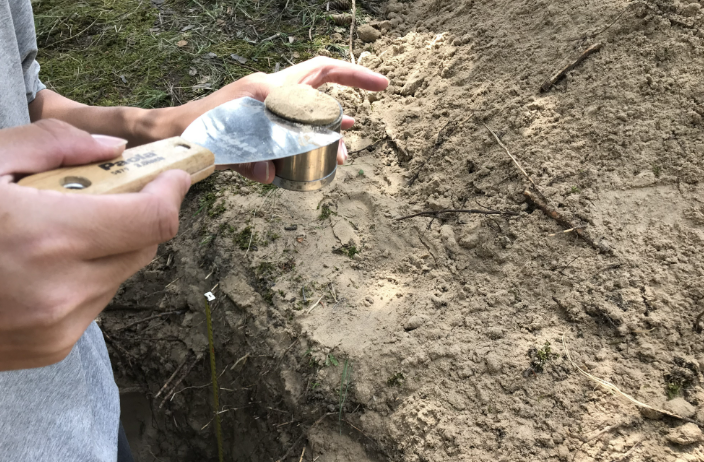

Supplement: Supplementary file 1 — Supplementary Information. [file 41598_2024_58259_MOESM1_ESM.zip › Raw data/Sample sampling site photos/1 (6).png]

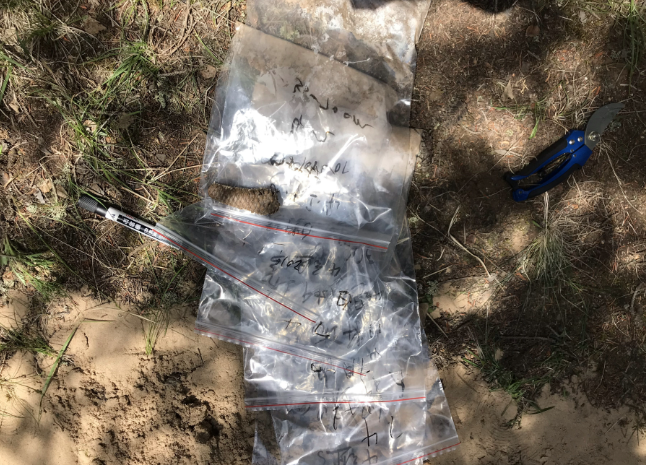

Supplement: Supplementary file 1 — Supplementary Information. [file 41598_2024_58259_MOESM1_ESM.zip › Raw data/Sample sampling site photos/1 (7).png]

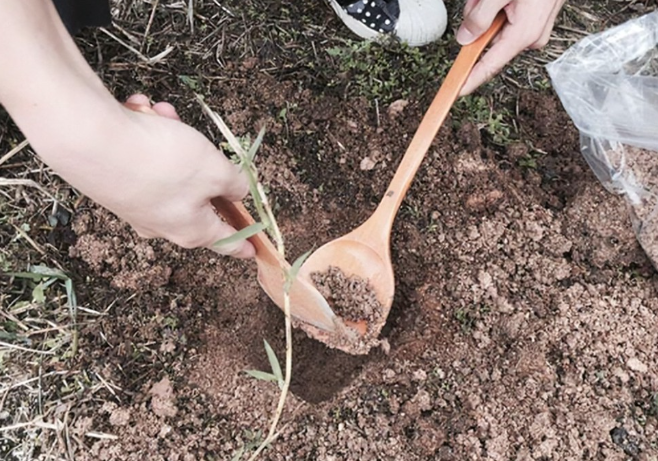

Supplement: Supplementary file 1 — Supplementary Information. [file 41598_2024_58259_MOESM1_ESM.zip › Raw data/Sample sampling site photos/1 (8).png]

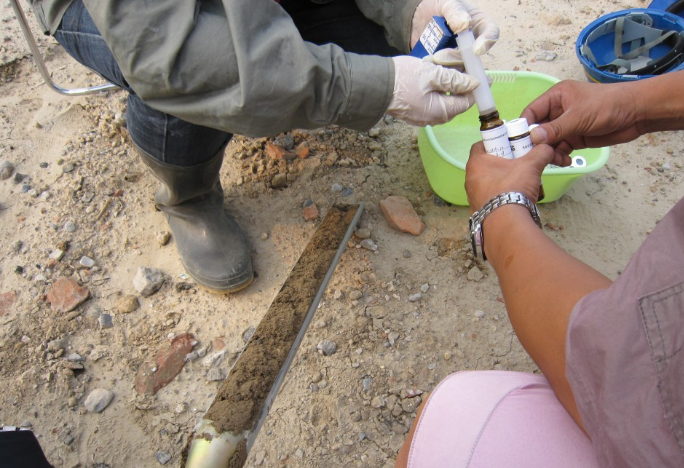

Supplement: Supplementary file 1 — Supplementary Information. [file 41598_2024_58259_MOESM1_ESM.zip › Raw data/Sample sampling site photos/1 (9).png]
